# Cardiac Optimisation in Proximal Femoral Fractures in the Elderly

**DOI:** 10.7759/cureus.91027

**Published:** 2025-08-26

**Authors:** Sadhin Subhash, Paul Njoku, Shahtajarab Saltanat, Mohammed F Millat, Sayam Subhash, Sarmila Tharmakulasingam, Maheswaran W Archunan, David T Loveday, George Smith, Ignatius Liew, Sebastian Ho

**Affiliations:** 1 Trauma and Orthopaedics, East Suffolk and North Essex NHS Foundation Trust, Ipswich, GBR; 2 Cardiology, Frimley Health NHS Foundation Trust, Ascot, GBR; 3 Trauma and Orthopaedics, Broomfield Hospital, Chelmsford, GBR; 4 Physiology, Government Medical College, Kollam, IND; 5 Oncology, Norfolk and Norwich University Hospitals NHS Foundation Trust, Norwich, GBR; 6 Trauma and Orthopaedics, Norfolk and Norwich University Hospitals NHS Foundation Trust, Norwich, GBR; 7 Orthopaedics, Norfolk and Norwich University Hospitals NHS Foundation Trust, Norwich, GBR; 8 Trauma and Orthopaedic Surgery, Addenbrooke's Hospital, Cambridge University Hospitals NHS Foundation Trust, Cambridge, GBR

**Keywords:** acute and chronic heart failure, cardiac disease, fragility hip fractures, orthogeriatric medicine, orthopaedic surgery, pacemaker, perioperative acute myocardial infarction, post-operative atrial fibrillation

## Abstract

Fragility hip fractures are increasingly common in elderly patients and are associated with high morbidity and mortality. Cardiovascular comorbidities-including ischaemic heart disease, heart failure, and valvular disease-contribute significantly to poor outcomes. This study aimed to review perioperative cardiac complications in elderly patients undergoing fragility hip fracture repair and evaluate strategies for optimisation, with emphasis on postoperative atrial fibrillation (POAF), acute myocardial infarction (AMI), heart failure, and management of cardiac implantable electronic devices (CIEDs).

We conducted a narrative review drawing data from the databases PubMed, Embase, and the Cochrane Library (January 2000-March 2024). The search terms included “hip fracture,” “cardiac complications,” “postoperative atrial fibrillation,” “myocardial infarction,” “heart failure,” “valvular disease,” and “cardiac implantable electronic devices.” Guidelines from the National Institute for Health and Care Excellence (NICE), the European Society of Cardiology (ESC), and the American College of Cardiology/American Heart Association (ACC/AHA) were also reviewed.

POAF was observed in ~3-4% of elderly hip fracture patients and is associated with significantly higher one-year mortality (60% vs. 19.5%). Risk factors include surgical delay beyond 48 hours and transfusion of >2 units of packed red blood cells. AMI and perioperative heart failure are frequently underdiagnosed due to atypical presentations. CIED management requires multidisciplinary coordination to avoid device malfunction.

Cardiac optimisation in fragility hip fracture patients remains challenging due to heterogeneous evidence and variable practice. Development of validated POAF risk prediction tools, standardised treatment protocols, and structured multidisciplinary pathways may help improve outcomes and reduce healthcare burden.

## Introduction and background

Fragility hip fractures refer to fractures of the femur produced by low-energy trauma, such as falls from a vertical height. With the significant increase in the ageing population, falls are common, and to prevent further deterioration of health, a timely orthopaedic surgery is required. In the United Kingdom alone, the National Hip Fracture Database reports more than 66,000 cases annually, placing a significant burden on the National Health Service (NHS). These fractures are associated with a 30-day mortality rate of approximately 6-10%, and one-year mortality can exceed 25%. Postoperative atrial fibrillation (POAF) is considered to be the most common postoperative tachyarrhythmia in patients with fragility hip fractures and is linked to an increased risk of morbidity and mortality [1-3]. Fragility hip fractures have been strongly associated with ischemic heart disease [4,5], heart failure [4,6-8], and peripheral artery disease [4,9].

As a causal relationship between cardiovascular disease and fragility hip fractures has been suggested by several authors [4,6,10], preoperative management of cardiovascular disease involves cardiologists. However, cardiac disease occurring in the perioperative and postoperative period needs further attention and research to reduce the enormous burden on the NHS, as well as morbidity and mortality related to the orthopaedic surgeries. Cardiovascular comorbidities are also a leading contributor to poor perioperative outcomes and higher complication rates [11,12]. To further elucidate the optimal cardiac management, we reviewed the relevant literature on peri and postoperative fragility hip fracture-related cardiovascular complications and their management.

## Review

This narrative review was conducted to synthesise existing evidence on perioperative cardiac complications in elderly patients undergoing proximal femoral fracture repair. A narrative approach was chosen due to the heterogeneity of available literature, which spans multiple cardiovascular conditions and diverse clinical contexts. A literature search was performed on the databases PubMed, Embase, and the Cochrane Library for articles published between January 2000 and March 2024. The search strategy combined the terms "hip fracture, cardiac complications, postoperative atrial fibrillation, myocardial infarction, heart failure, valvular disease, and cardiac implantable electronic devices" using Boolean operators (AND/OR). Medical Subject Headings (MeSH) were applied where appropriate.

The inclusion criteria were as follows: (i) peer-reviewed studies in English, (ii) studies involving patients aged ≥65 years, and (iii) studies focusing on perioperative cardiac complications in hip fracture surgery. Guidelines and expert consensus documents from recognised professional bodies were also included. The exclusion criteria were as follows: (i) case reports, conference abstracts, and non-peer-reviewed sources, and (ii) studies limited to elective orthopaedic procedures or non-cardiac complications.

Guidelines from the National Institute for Health and Care Excellence (NICE), the European Society of Cardiology (ESC), and the American College of Cardiology/American Heart Association (ACC/AHA) were reviewed to provide evidence-based context. Given the heterogeneity of study designs and outcomes, no quantitative meta-analysis was performed.

Results

A review of the literature highlights several perioperative cardiovascular complications in elderly patients with fragility hip fractures. These include POAF, perioperative myocardial infarction (AMI), acute and chronic heart failure, valvular heart disease, and issues related to cardiac implantable electronic devices (CIEDs). Table 1 summarises the management strategies for these perioperative cardiac complications.

**Table 1:** Management strategies for perioperative cardiac complications Strategies classified by pharmacologic and non‑pharmacologic interventions, and distinguishing approaches for stable versus unstable patients ACE: angiotensin-converting enzyme; CIED: cardiac implantable electronic device; ECG: electrocardiogram; ICD: implantable cardioverter-defibrillator; PCI: percutaneous coronary intervention

Management strategies			
Postoperative atrial fibrillation (POAF)	Hemodynamically stable	Beta-blockers or calcium channel blockers for rate control. Anticoagulation guided by CHA₂DS₂‑VASc and ORBIT scores.	Correct electrolyte disturbances. Early mobilisation. Avoid delays to surgery (>48 hours increases POAF risk; OR: 1.66)
	Unstable (hypotension, ischaemia)	Urgent electrical cardioversion. Intravenous amiodarone	
Continuous ECG monitoring. Protocol-based response (e.g., anaesthetic/cardiology input)			
Acute myocardial infarction (AMI)	All patients	Aspirin followed by dual antiplatelet therapy. Statin, beta‑blocker, and ACE inhibitor	Early ECG and troponin surveillance. Early cardiology review and consideration of PCI
Heart failure	Chronic or decompensated	Diuretics as needed. Optimisation of heart failure medications (e.g., ACE inhibitors, beta‑blockers)	Preoperative echocardiography if no recent imaging. Fluid balance monitoring. Multidisciplinary co‑management
Valvular disease (e.g., aortic stenosis)	Symptomatic or high-risk features	Manage hypertension or heart failure symptoms pharmacologically	Preoperative echocardiography when indicated. Expedite surgery (avoid unnecessary delays)
CIEDs (pacemakers/ICDs)	All cases	Ensure medications (e.g., rate controllers) are optimised	Notify anaesthetics and cardiology teams. Deactivate ICD if appropriate. Plan perioperative device interrogation

Postoperative Atrial Fibrillation (POAF)

POAF is the most common tachyarrhythmia following hip fracture surgery, typically occurring within 48-96 hours postoperatively [1]. It is a recognised complication of hip fracture repair in elderly patients [2]. Other postoperative complications include infection, blood loss, and neurovascular injury; however, POAF is frequently underestimated as it was previously considered a transient arrhythmia with favourable outcomes. More recent evidence demonstrates that POAF is associated with prolonged hospitalisation, increased morbidity, and higher mortality [2,3]. The pathophysiology of POAF is multifactorial. The contributing mechanisms include (i) the physiological stress response to surgery [4], (ii) autonomic activation from adrenergic stimulation and systemic inflammation, and (iii) increased sympathetic tone in the postoperative state [5]. Acute management of POAF is based on four key principles: (1) rate or rhythm control, (2) haemodynamic and symptom stabilisation, (3) correction of underlying precipitants, and (4) prevention of recurrence and thromboembolic complications [6]. NICE guidelines recommend that POAF after non-cardiothoracic surgery should be managed in the same way as new-onset atrial fibrillation of any other cause [7].

Initial management requires clinical assessment and correction of reversible triggers. Treatment depends on haemodynamic stability; unstable patients (with ischaemia, syncope, or acute heart failure) require emergency DC cardioversion with cardiology input. Pharmacological cardioversion using amiodarone or flecainide may be considered at the cardiologist’s discretion. Stable patients should receive rate control, typically with beta-blockers or non-dihydropyridine calcium channel blockers (e.g., verapamil). In patients with heart failure, amiodarone or digoxin may be more appropriate. All patients should undergo thromboembolic risk assessment using CHA₂DS₂-VASc [8], balanced against bleeding risk assessed by tools such as the ORBIT score, as recommended by NICE [7].

POAF presents diagnostic and management challenges due to its variable presentation. At present, no validated tool exists to reliably identify patients at the highest risk following hip fracture surgery. The development of a prediction model would allow closer monitoring and proactive management of high-risk patients. Recommended preventive measures include continuous cardiac monitoring for 24-48 hours postoperatively [5], correction of electrolyte imbalances (particularly potassium and magnesium), assessment of thyroid function, and careful management of blood pressure and fluid balance. Adequate postoperative analgesia (e.g., patient-controlled analgesia or epidurals) is also essential, as uncontrolled pain and nociception can trigger POAF.

In summary, management of POAF after hip fracture repair focuses on preventing cardioembolic complications with anticoagulation and controlling ventricular rate or restoring sinus rhythm. Long-term anticoagulation is recommended in accordance with NICE guidance, even if the arrhythmia resolves. Specialist cardiology input should guide decisions on rhythm control and the duration of therapy. Outpatient cardiology follow-up, typically at 6-12 months, is appropriate for patients with recurrent or refractory POAF, thromboembolic complications, coexisting cardiac disease (e.g., heart failure), or persistent symptomatic AF. Hospitals and local trusts should consider developing systematic management algorithms to ensure effective in-hospital treatment and structured follow-up of patients with POAF.

Cardiac Implantable Electronic Devices

Management of patients with CIEDs, such as pacemakers and implantable cardioverter defibrillators (ICDs), requires coordinated perioperative planning. Electromagnetic interference (EMI), particularly from monopolar diathermy, may lead to device malfunction, including inappropriate pacing inhibition or triggering of shocks. Where feasible, bipolar diathermy should be used, and grounding pads should be placed to direct current flow away from the device. Table 2 summarises the planning for CIEDs.

**Table 2:** Perioperative planning for CIEDs CIED: cardiac implantable electronic device; ECG: electrocardiogram; EMI: electromagnetic interference; ICD: implantable cardioverter-defibrillator

Phase	Considerations
Preoperative	Identify device type and indication. Contact cardiology for interrogation (if time allows). Determine pacing dependency. Plan ICD deactivation if required. Use bipolar diathermy when possible
Intraoperative	Minimise EMI exposure. Place the diathermy return pad away from the device. Monitor ECG and pulse oximetry continuously
Postoperative	Reactivate ICD (if applicable). Reassess device function. Arrange a formal follow-up interrogation if not performed preoperatively

Managing patients with pacemakers or ICDs in the context of emergency hip fracture surgery is complex. While routine preoperative interrogation may not be feasible, clinicians should remain vigilant for signs of device malfunction. Anaesthetists and cardiologists must be informed preoperatively, especially in patients with ICDs, as deactivation may be required.

Perioperative Acute Myocardial Infarction (AMI)

Hip fracture patients face an increased risk of coronary heart disease and AMI [13,14]. Perioperative myocardial infarctions are common yet frequently under-recognised, particularly in the very elderly, and are associated with poor outcomes [15,16]. Diagnosis is challenging due to atypical or silent presentations, with only about 15% of cases reporting chest pain. Clinical suspicion should be high in the presence of hypotension, ECG changes, or altered mental status. Early and serial ECGs with troponin measurement are critical for detection. Standard STEMI/NSTEMI management applies, including aspirin, beta-blockers, statins, and ACE inhibitors, with dual antiplatelet therapy considered based on bleeding risk. Selected patients may benefit from early coronary angiography or PCI, which may reduce in-hospital mortality in appropriate cases, although risks of bleeding and procedural delay must be weighed carefully [17].

Delaying hip fracture surgery for cardiac intervention carries significant risks. Surgical delays beyond 48 hours are associated with higher rates of infection, thromboembolic events, and mortality. For this reason, guidelines generally advise against postponing urgent hip fracture repair for an extensive cardiac workup unless absolutely necessary. Decisions should be individualised and made within a multidisciplinary team. Non-invasive coronary imaging, particularly coronary CT angiography, is under investigation as a rapid preoperative risk stratification tool in high-risk patients. It provides valuable anatomical information without the procedural risks of invasive angiography and may facilitate timely surgical decision-making. Although not yet standard practice, its potential role in urgent orthopaedic settings represents an important area for future research and protocol development.

Acute and Chronic Cardiac Failure

New-onset or decompensated cardiac failure is a common cause of hospital admissions in the elderly population. Heart failure remains one of the most common chronic cardiac conditions with an increasing incidence largely due to an ageing population [18]. Perioperative heart failure can complicate hip fracture surgery and is associated with a significantly increased length of hospital stay, morbidity and mortality in these patients [19].

**Figure 1:**
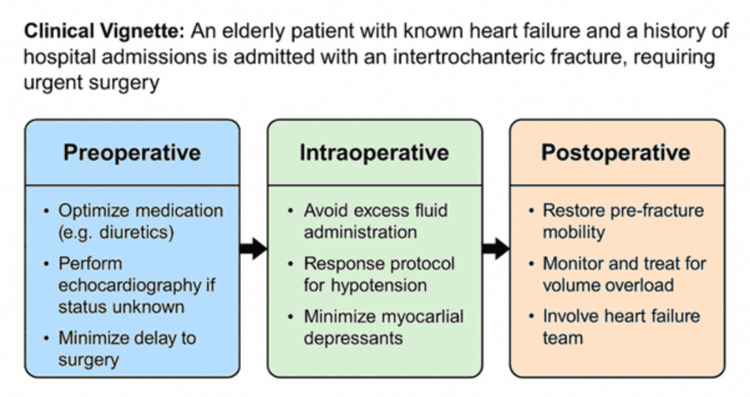
Flowchart for cardiac care in hip fragility fracture patients

ACC and AHA have published comprehensive guidelines on perioperative cardiovascular evaluation for patients undergoing non-cardiac surgery [20]. Although primarily designed for elective operations, these recommendations are also applicable to emergency procedures such as hip fracture repair. Patients with a history of heart failure and a recent change in clinical status should undergo an assessment of left ventricular (LV) function if this has not been performed within the past 12 months. Echocardiography remains the gold-standard imaging modality for assessing systolic and diastolic function [20]. Cardiac MRI is emerging as a complementary tool in evaluating cardiac function [21]. Echocardiography also provides information on valvular morphology, function, and structural heart disease.

Evidence consistently shows that delaying hip fracture surgery is harmful and worsens outcomes [22,23]. Early repair improves survival, reduces complications, and enhances long-term quality of life [24]. Surgical delay, by contrast, increases the risk of thromboembolism, pressure ulcers, loss of independence, and mortality. Therefore, the decision to pursue preoperative cardiac testing should be made carefully and guided by multidisciplinary discussion involving cardiology, anaesthesiology, and orthopaedics. For patients admitted with acute or decompensated heart failure, the recommended diagnostic workup includes: 12-lead ECG - to identify arrhythmias, myocardial ischaemia, ventricular hypertrophy, or strain. Chest X-ray - to evaluate cardiothoracic ratio, pulmonary congestion, and to assess response to therapy. BNP or NT-proBNP - elevated levels are highly sensitive for diagnosing heart failure. Echocardiography - to assess ventricular ejection fraction and confirm diagnosis.

Management of acute heart failure in the perioperative period focuses on symptom relief, reduction of fluid overload, and restoration of haemodynamic stability. Key interventions include oxygen therapy, ventilatory support when required, and intravenous diuretics to treat volume overload. Opiates may be used to relieve dyspnoea, while nitrates can help alleviate pulmonary congestion [20]. ESC has also published detailed guidelines on the management of acute heart failure [20]. While a full discussion is beyond the scope of this review, the principle remains that acute heart failure in hip fracture patients should be stabilised before surgical repair whenever possible. Given the high intraoperative and postoperative risks, adequate optimisation of the acute medical condition is essential to achieve the best possible surgical outcomes.

Valve Disease and New Heart Murmurs

Aortic stenosis, most often secondary to senile calcification, is the most common acquired valvular pathology in the elderly [25]. Its prevalence continues to rise with ageing populations, particularly in developed countries. As a result, concomitant valve disease is frequently seen in orthogeriatric patients presenting with fragility hip fractures. Clinical presentation varies widely, ranging from an incidental finding in asymptomatic patients to severe decompensated heart failure. Echocardiography remains the gold standard for investigating systolic murmurs suggestive of aortic stenosis or mitral regurgitation [26]. The perioperative management of aortic stenosis in elderly hip fracture patients is particularly challenging. Anaesthetic concerns include the need to maintain haemodynamic stability intraoperatively and the increased risk of perioperative arrhythmias. While preoperative echocardiography can provide valuable information, it is consistently associated with surgical delays. Multiple studies demonstrate that delays due to preoperative imaging are linked to higher morbidity and mortality [27-29]. Early hip fracture repair, by contrast, improves survival, reduces complications, and preserves independence [24].

Decisions about pursuing echocardiography should therefore be made on a case-by-case basis, balancing the risks of delay against potential anaesthetic benefit. In most cases, the severity of aortic stenosis does not significantly alter anaesthetic management during hip fracture repair [30]. Preoperative echocardiography should be reserved for patients with red-flag clinical features, including syncope, angina, evidence of decompensated heart failure, or ECG findings of ventricular hypertrophy and strain. Routine echocardiography after the incidental detection of a murmur should be avoided in the emergency setting. Symptomatic patients (e.g., chest pain, exertional syncope, orthopnoea, or decompensated heart failure) should undergo transthoracic echocardiography (TTE) to confirm valve severity and guide perioperative risk management. Asymptomatic patients with stable clinical status and no high-risk features may proceed to surgery without delay, especially in urgent cases. Overall, the default approach should be to prioritise timely surgery, reserving preoperative cardiac imaging for patients with clear clinical indications.

Discussion

Optimising cardiovascular care in elderly patients undergoing surgery for fragility hip fractures is crucial to improving survival and functional outcomes. Moreover, the paper determines that current literature assists the relationship between hip fractures and an increased mortality along with an augmented risk of AMI [13,14]. It states the fact that the medical management of AMIs within candidates for hip fracture healing is highly determined by professional views in analysing the timing of delay to surgery and coronary revascularisation [15,16]. Meanwhile, the paper demonstrates the fact that the peri-operative organisation of antiplatelets should take into account the possibility of thrombotic events as a result of episodic treatment in comparison with the related bleeding risk of surgery, with some evidence suggesting a potential role for early coronary angiography in selected patients [17].

Contradictory Evidence and Emerging Practices

There is a growing recognition that many cardiovascular complications in this population-such as POAF and myocardial injury-are underdiagnosed due to atypical presentations and limited postoperative monitoring. However, evidence remains mixed on whether proactive interventions (e.g., routine postoperative troponin screening or expanded use of CT angiography) improve outcomes or simply increase resource use. Likewise, the role of invasive procedures such as PCI in acute perioperative MI remains debated, particularly given the risks of delaying hip surgery in frail patients.

Gaps in Guideline Implementation

Despite existing frameworks, real-world uptake of multidisciplinary cardiac optimisation pathways remains inconsistent. Many institutions lack rapid access to preoperative echocardiography, CIED interrogation, or specialist input, especially out of hours. This results in ad hoc decision-making, which may not align with best practices. Emerging models such as shared orthogeriatric-cardiology care, point-of-care ultrasound (POCUS), and embedded clinical decision tools may help bridge this gap, but remain underutilised.

Limitations of This Review

This review is narrative rather than systematic, and no formal risk of bias assessment or meta-analysis was conducted. As such, it may be prone to selection bias and cannot provide quantitative effect estimates. Additionally, many studies included were observational or retrospective in nature, limiting their generalisability. The review also does not address cost-effectiveness or the feasibility of implementing proposed strategies in resource-constrained settings.

## Conclusions

This review highlights the importance of optimising cardiac care in patients with fragility hip fractures. POAF remains under-recognised despite its strong association with increased morbidity and mortality. Current NICE guidance advises managing POAF in the same way as new-onset atrial fibrillation after non-cardiothoracic surgery; however, patients with refractory arrhythmias may ultimately require device therapy, such as ICDs or permanent pacemakers. Hip fracture patients are also at substantially increased risk of AMI, with a higher incidence of coronary heart disease and myocardial infarction following fracture. Perioperative management often requires balancing the risks of delaying surgery against the potential benefits of revascularisation, while antiplatelet therapy must be carefully tailored to minimise both thrombotic and bleeding risks. Similarly, heart failure is a frequent perioperative complication, linked to longer hospital stays and worse outcomes. Multidisciplinary input from cardiology, anaesthetics, and orthopaedics is therefore essential to guide perioperative decision-making and to optimise outcomes. The development of validated risk stratification tools and structured perioperative management pathways is urgently needed to reduce postoperative cardiovascular complications, improve survival, and enhance recovery and quality of life in this vulnerable population.
